# Acknowledgment to Reviewers of *Journal of Cardiovascular Development and Disease* in 2020

**DOI:** 10.3390/jcdd8020007

**Published:** 2021-01-22

**Authors:** JCDD Editorial Office

**Affiliations:** MDPI AG, St. Alban-Anlage 66, 4052 Basel, Switzerland

Peer review is the driving force of journal development, and reviewers are gatekeepers who ensure that *Journal of Cardiovascular Development and Disease* maintains its standards for the high quality of its published papers. Thanks to the cooperation of our reviewers, in 2020, the median time to first decision was 20.5 days and the median time to publication was 42 days. The editors would like to express their sincere gratitude to the following reviewers for their precious time and dedication, regardless of whether the papers were finally published:
Adler, EricKruithof, BoudewijnAhmad, ShaadKurath-Koller, StefanAiello, VeraKutikhin, Anton G.Amack, JeffreyKuwata, ShingoAmri, Ibtihal AlKwon, Yu-jinAnderson, RobertLatif, NajmaAngelini, PaoloLee, Wei-ChiehAstiazaran-Garcia, HumbertoLeire, UnzuéAzhar, MohamadLevi, GiovanniBakkers, Jeroen P.W.Lin, WenBaldini, AntonioLu, QuanlongBallmann, ChristopherLynge, Thomas HadbergBamforth, Simon D.Ma, HongBarrett, HughMachon, OndrejBelo, José AntónioMänner, JörgBeyer, Eric C.Mariotti, AlessandroBittel, Douglas C.Markovic, SinisaBogomolovas, JulijusMarrs, James A.Borghini, AndreaMarsan, Nina AjmoneBrčić Karačonji, IrenaMasarone, DanieleBronner, MarianneMasella, RobertaBuch, EricMaslen, Cheryl L.Buchholz, JohnMauro, FeolaCabezas, Jose MonteroMavrogeni, SophieCalissendorff, JanMessonnier, Laurent A.Calvo, DavidMestroni, LuisaCampione, MarinaMetzinger, LaurentCampuzano, OscarMeuris, BartCapoccia, MassimoMiyachi, HidekiCaroleo, Maria CristinaMiyagawa-Tomita, SachikoCasini, AlessandroMohsin, SadiaCeolotto, GiulioMongillo, MarcoChaudhry, BillMorrow, Bernice E.Chen, ChaoMozos, IoanaChen, Jau-nianNorris, RussellChevret, EdithPanayiotou, Andrie G.Ciccone, MarcoPekkan, KeremCollins, Michelle M.Pepi, MauroColussi, Gian LucaPoelmann, RobertConway, SimonPrickett, TimCornell, RobertPrzybylowicz, KatarzynaCrocini, ClaudiaPuślecki, MateuszEller, KathrinRauch, BernhardEzhov, MaratRugonyi, SandraFaletra, FrancescoRuiz, SantiagoFavaloro, Emmanuel J.Sampietro, TizianaFukunaga, NaotoSarecka-Hujar, BeataGalatou, EleftheriaSato, AkiraGarcia-Jares, CarmenScambler, PeterGatzoulis, Konstantinos A.Schlüter, Klaus-DieterGentile, MarcoSchoenhagen, PaulGiordano, RaffaeleSenesi, PamelaGiovannoni, RobertoShiraishi, IsaoGomez-Sanchez, EliseShiratori, HidetakaGorini, FrancescaStapleton, PhoebeGragnano, FeliceStefani, LauraGrau, MarijkeStoffman, JaysonGroot, A.C. Gittenberger-deSuñer, Damian HeineHafiane, AnouarSurdacki, AndrzejHarenberg, Job F.Tan, Bee KangHenderson, DeborahThomas, ShaneHiebert, LindaToma, MilanHolaj, RobertTranter, MichaelHolzmann, KlausTretter, Justin T.Horowitz, John D.Tsang, MichaelHouyel, LucileTseng, Elaine E.Hsu, EdwardTuriel, MaurizioHwang, Chueh-LungVan Heesch, SebastiaanIchibori, YasuhiroVancheri, FedericoImamura, TeruhikoVaškelytė, JolantaIrwin, David C.Vecoli, CeciliaIshibashi, NobuyukiVelasco, Carlos E.Jänig, WilfridWang, JiongweiJaźwińska, AnnaWang, XuVan Den Hoff, MauriceWatanabe, EiichiJean Francois, SchvedWessells, RobertJensen, BjarkeWigle, JeffreyJiao, KaiWilczek, PiotrJinnouchi, HiroyukiWingert, RebeccaJohn, PepperXin, MeiJulius, UlrichXu, XiaoleiKasai, TakatoshiYang, ZhongzhouKeller, BradleyYutzey, KatherineKelly, RobertŽáček, PavelKocica, Mladen J.Zaffran, StephaneKostner, Gert M.Zhang, JianyiKowalski, William J.


